# Bidirectional Dysregulation of AMPA Receptor-Mediated Synaptic Transmission and Plasticity in Brain Disorders

**DOI:** 10.3389/fnsyn.2020.00026

**Published:** 2020-07-10

**Authors:** Hongyu Zhang, Clive R. Bramham

**Affiliations:** Department of Biomedicine, University of Bergen, Bergen, Norway

**Keywords:** AMPA receptor (AMPAR), synaptic transmission and plasticity, AMPAR trafficking, neurodegenarative diseases, neuropsychiatric disorders, neurodevelopmental disorders

## Abstract

AMPA receptors (AMPARs) are glutamate-gated ion channels that mediate the majority of fast excitatory synaptic transmission throughout the brain. Changes in the properties and postsynaptic abundance of AMPARs are pivotal mechanisms in synaptic plasticity, such as long-term potentiation (LTP) and long-term depression (LTD) of synaptic transmission. A wide range of neurodegenerative, neurodevelopmental and neuropsychiatric disorders, despite their extremely diverse etiology, pathogenesis and symptoms, exhibit brain region-specific and AMPAR subunit-specific aberrations in synaptic transmission or plasticity. These include abnormally enhanced or reduced AMPAR-mediated synaptic transmission or plasticity. Bidirectional reversal of these changes by targeting AMPAR subunits or trafficking ameliorates drug-seeking behavior, chronic pain, epileptic seizures, or cognitive deficits. This indicates that bidirectional dysregulation of AMPAR-mediated synaptic transmission or plasticity may contribute to the expression of many brain disorders and therefore serve as a therapeutic target. Here, we provide a synopsis of bidirectional AMPAR dysregulation in animal models of brain disorders and review the preclinical evidence on the therapeutic targeting of AMPARs.

## Introduction

**Figure 1:**
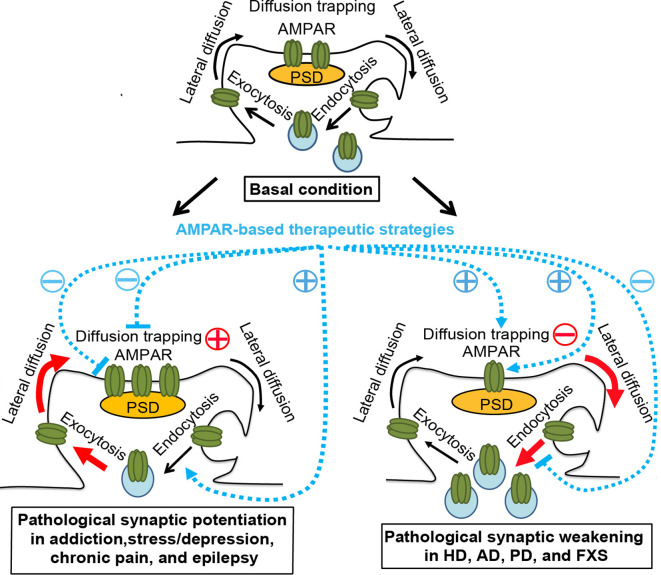
AMPAR trafficking under physiological and pathological conditions and AMPAR-based therapeutic strategies. **Top panel**, basal condition. AMPARs constitutively cycle between intracellular pools and the neuronal surface *via* endocytosis and exocytosis. At the plasma membrane, AMPARs bidirectionally exchange between extrasynaptic and synaptic compartments by lateral diffusion, powered by thermal agitation. This is readily perturbed by protein-protein interactions at postsynaptic sites, where AMPARs are trapped by reversible binding to postsynaptic density (PSD) proteins, cytoskeletal proteins, adhesion proteins, or extracellular matrix. The dynamic equilibrium established between different pools allows the steady state levels of AMPARs at synapses. **Bottom left panel**, pathological conditions, such as addiction, stress/depression, chronic pain, and epilepsy. The equilibrium is shifted towards the accumulation of postsynaptic AMPARs in a region/circuit-specific and subunit-specific manner. This may be due to the enhanced diffusion trapping mechanisms and/or potentiated exocytosis (Red). AMPAR lateral diffusion mediates the recruitment of extrasynaptic AMPARs to the postsynaptic membrane. AMPAR-based therapeutic strategies, which ameliorate drug-seeking behavior, chronic pain, or epileptic seizures in corresponding animal models, include reducing diffusion trapping and/or enhancing endocytosis (such as with optogenetic LTD induction or mGluR1 positive allosteric modulator), or inhibiting AMPAR-mediated currents by AMPAR antagonists. **Bottom right panel**, pathological conditions, such as Huntington’s disease (HD), Alzheimer’s disease (AD), Parkinson’s disease (PD), and fragile X syndrome (FXS). The equilibrium is shifted towards the dispersal of AMPARs from the postsynaptic membrane in hippocampal and cortical neurons, which is linked to cognitive deficits, likely through the impairment of diffusion trapping mechanisms resulting in an increase in AMPAR lateral diffusion, potentiation of endocytosis, and/or suppression of exocytosis (Red). AMPAR-based therapeutic strategies, which improve synaptic plasticity and/or memory in animal models of HD, AD and FXS, include enhancing diffusion trapping mechanisms (such as with tianeptine), blocking endocytosis (such as with mGluR5 antagonists), or enhancing AMPAR function (such as with AMPAR positive allosteric modulators)(Blue). However, the effects of these strategies in human patients remain to be determined. Brain region- and AMPAR subunit-specific treatment is needed.

Synaptic plasticity is central to memory and other adaptive responses of adult neural circuits. NMDA receptor (NMDAR)-dependent long-term potentiation (LTP) and long-term depression (LTD) are triggered by the activation of NMDARs but expressed by an increase or decrease in the abundance of AMPA receptors (AMPARs) at the postsynaptic membrane, respectively. Postsynaptic LTD induced by the activation of group I metabotropic glutamate receptors (mGluR-LTD) is similarly expressed by a reduction in the number of postsynaptic AMPARs (Luscher and Huber, 2010). AMPARs are tetrameric complexes composed of GluA1, GluA2, GluA3, or GluA4 subunits, with GluA1/2 heteromers dominant at hippocampal CA1 synapses. The number and composition of postsynaptic AMPARs are in a dynamic balance, which is achieved by AMPAR trafficking. AMPAR trafficking involves intracellular transport, endo-/exo-cytosis, recycling, lateral surface diffusion, and degradation (Choquet, 2018). Newly synthesized receptors are transported intracellularly on microtubules from soma to dendrites. Through exocytosis/endocytosis, AMPARs cycle between intracellular and surface pools. Recycling refers to the process by which endocytosed receptors are returned to the cell surface *via* exocytosis. Surface AMPARs exchange between synaptic and extrasynaptic compartments *via* lateral diffusion and are reversibly trapped at synapses by postsynaptic scaffold proteins, cytoskeletal proteins, adhesion proteins, or extracellular matrix. This continuous exchange of receptors between different pools establishes a dynamic equilibrium (Figure 1, top panel). This balance can be shifted in response to neuronal/synaptic activity (Opazo and Choquet, 2011). For example, during LTP, AMPARs are selectively recruited, by lateral diffusion, to the postsynaptic membrane to increase synaptic strength, while exocytosed AMPARs serve as an extrasynaptic reservoir (Makino and Malinow, 2009; Penn et al., 2017). Conversely, during LTD, AMPARs are dispersed, through endocytosis, from the postsynaptic membrane to reduce synaptic transmission. Thus, synaptic strength at single synapses is bidirectionally regulated *via* AMPAR trafficking.

Increasing evidence shows that learning and memory can be modified at the cellular and molecular levels by acute modulation of LTP, LTD, or AMPAR trafficking. For example, fear memory established by associating a foot-shock with optogenetic stimulation of auditory inputs to the amygdala was inactivated by subsequent optogenetic delivery of LTD to the conditioned auditory input and further reactivated by optogenetic delivery of LTP (Nabavi et al., 2014). Moreover, PhotonSABER, an optogenetic tool developed to inhibit AMPAR endocytosis during LTD in a light-dependent manner, was applied in Purkinje cells and inhibited cerebellar motor learning (Kakegawa et al., 2018). Furthermore, immobilization of surface AMPARs by crosslinking approaches markedly impaired hippocampal LTP *in vivo* and also inhibited contextual fear conditioning (Penn et al., 2017). These results support a causal contribution of AMPAR trafficking and synaptic plasticity to learning and memory. Second, they suggest that learning and memory can be manipulated at the molecular level (AMPARs trafficking), as well as at the cellular level by induction of LTP or LTD.

Notably, the dynamic equilibrium of AMPAR trafficking can also be shifted under pathological conditions (Figure 1, bottom left and bottom right panels). Increasing evidence suggests that brain region-specific and AMPAR subunit-specific aberrant enhancement or reduction in synaptic transmission or plasticity occurs with many neurodegenerative, neurodevelopmental, and neuropsychiatric disorders. A reversal of these aberrations ameliorates drug-seeking behavior, chronic pain, epileptic seizures, or cognitive deficits. Although the etiology, pathogenesis, and symptoms vary greatly between disorders, the observed restoration of function and mitigation of symptoms suggest that dysregulation of AMPAR-mediated synaptic transmission and plasticity is a convergence point for multiple pathological pathways, rather than a compensatory protective mechanism.

Below, we review the evidence of brain region-specific and AMPAR subunit-specific bidirectional dysregulation of AMAPR-mediated synaptic transmission and plasticity in animal models of several of the most common neurodegenerative, neurodevelopmental and neuropsychiatric disorders. We also review the therapeutic effects exerted by targeting AMPAR subunits or trafficking. We propose that, despite the highly diverse underlying pathologies, dysregulation of AMPARs is a common mechanism in the expression of different diseases, and bidirectional therapeutic targeting of AMPARs may be a promising mitigation strategy for various diseases. Lastly, we discuss the potential therapeutic application of small interfering peptides and aptamers, as well as the need for new optogenetic and optopharmacological tools for elucidating the molecular mechanisms and spatial-temporal dynamics of AMPA regulation in animal models of brain disorders.

### Addiction

Addiction is a psychological and physical inability to stop consuming a chemical, despite adverse consequences. Many addictive drugs induce changes in the synaptic composition of AMPARs and alter synaptic plasticity within reward circuitry, such as the ventral tegmental area (VTA), nucleus accumbens (NAc), prefrontal cortex (PFC), dorsal medial striatum (DMS), and amygdala (Ungless et al., 2001; Saal et al., 2003; Wolf, 2016; Cooper et al., 2017). These drugs include cocaine (Conrad et al., 2008), delta(9)-tetrahydrocannabinol (Good and Lupica, 2010), methamphetamine (Scheyer et al., 2016), amphetamine (Saal et al., 2003), benzodiazepines (Tan et al., 2010), nicotine (Marchi et al., 2015), morphine (Madayag et al., 2019), heroin (Van den Oever et al., 2008), and alcohol (Ma et al., 2018). For instance, cocaine exposure or self-administration induced silent synapses, which do not contain AMPARs but only NMDARs, in NAc (Ma et al., 2014; Huang et al., 2015). However, extended withdrawal (e.g., 30–45 days) from cocaine self-administration resulted in the unsilencing of synapses *via* the synaptic insertion of Ca^2+^-permeable (CP) GluA2-lacking AMPARs. This indicated that CP-AMPARs do not primarily mediate drug-seeking *per se*, but rather contribute to withdrawal-dependent enhancement (incubation) of cocaine-seeking behavior (Conrad et al., 2008; Lee et al., 2013; Ma et al., 2014; Scheyer et al., 2016). Optogenetically induced LTD resulting in CP-AMPAR removal from amygdala-to-NAc synapses attenuated incubation of cocaine craving (Lee et al., 2013). Besides, the application of a mGluR1 positive allosteric modulator, by removing CP-AMPARs from NAc synapses, also reduced the expression of incubated cocaine craving (Loweth et al., 2014). Further evidence suggested that, depending on the input pathway, synaptic insertion of non-CP-AMPARs also underlies cocaine-seeking behavior. In one study, extended cocaine withdrawal evoked the insertion of CP-AMPARs in NAc synapses receiving input from medial PFC, whereas non-CP-AMPARs were inserted at synapses with input from the ventral hippocampus. Optogenetic reversal of plasticity at both inputs abolished cocaine seeking (Pascoli et al., 2014). In another study, extended cocaine withdrawal induced CP-AMPAR insertion in NAc synapses with infralimbic (IL) mPFC input, whereas non-CP-AMPARs were inserted at synapses with prelimbic (PrL) mPFC input. Optogenetic reversal of the plasticity of IL-to-NAc and PrL- to-NAc projections enhanced and reduced, respectively, incubation of cocaine craving (Ma et al., 2014), suggesting a circuit-dependent mechanism. Moreover, optogenetic LTP and LTD induction at projections from mPFC to DMS increased and decreased alcohol-seeking behavior, respectively (Ma et al., 2018). Furthermore, microinjection of NASPM, a synthetic analog of Joro spider toxin that selectively inhibits homomeric GluA1-AMPARs (CP-AMPARs), into the central nucleus of the amygdala, reduced morphine intake of rats (Hou et al., 2020).

Clathrin- and GluA2-dependent AMPAR endocytosis appears to play a crucial role in D-amphetamine-induced behavioral sensitization (Brebner et al., 2005; Choi et al., 2014), morphine-induced place preference (Dias et al., 2012) and cue-induced reinstatement of heroin self-administration (Van den Oever et al., 2008). Blocking regulated AMPAR endocytosis and LTD by GluA2-derived peptide (Tat-GluA2_3Y_) prevented the expression and maintenance of D-amphetamine-induced behavioral sensitization (Brebner et al., 2005; Choi et al., 2014), facilitated the extinction of morphine-induced conditioned place preference (Dias et al., 2012), and reduced heroin seeking (Van den Oever et al., 2008). Collectively, these results suggest a critical role for AMPAR trafficking and AMPAR-mediated plasticity in addictive behavior. Targeting AMPAR subunits or AMPAR trafficking may represent a therapeutic strategy for addiction.

### Stress/Depression

Stress in humans is defined as bodily or mental tension caused by physical, mental, or emotional factors.

Stressor exposure promotes the release of hormones from the adrenal gland, such as corticosterone, epinephrine, and norepinephrine (NE). The effect of stress on AMPAR synaptic plasticity is complex and region-specific (McGrath and Briand, 2019). For example, the administration of corticosterone to hippocampal neuronal cultures resulted in time-dependent synaptic accumulation of GluA2-AMPARs, possibly by increasing GluA2-AMPAR surface diffusion (Groc et al., 2008). The regulation of AMPARs was biphasic, with mineralocorticoid receptors (MRs) and glucocorticoid receptors (GRs) mediating early (minutes) and late (hours) responses to corticosterone, respectively. The MR-dependent increase in synaptic AMPAR contents facilitated chemical LTP induction, while the GR-dependent increase occluded chemical LTP (Groc et al., 2008; Krugers et al., 2010). This reflected a saturation process of LTP and also provided a cellular mechanism for the finding that short bath application of corticosterone (100 nM, 5–10 min) to hippocampal slices enhanced the frequency of AMPAR miniature excitatory postsynaptic potentials (mEPSC) in CA1 pyramidal neurons *via* MRs (Karst et al., 2005), while within hours, corticosterone slowly increased the amplitude of AMPAR mEPSC through GRs (Karst and Joëls, 2005) and impaired synaptic potentiation (Kim and Diamond, 2002; Zhang et al., 2013). Notably, the antidepressant tianeptine reversed the corticosterone-induced increase in AMPAR surface diffusion in hippocampal neurons and restored hippocampal LTP in slices from acutely stressed mice (Zhang et al., 2013). This suggests that reversal of AMPAR surface trafficking may contribute to the restoration of hippocampal synaptic plasticity in animal models of stress. Other stressful stimuli, such as NE and emotional stress, induced the phosphorylation and synaptic delivery of GluA1-AMPARs in hippocampal slice cultures, which was thought to lower the threshold for LTP (Hu et al., 2007). It is noteworthy that social defeat stress was shown to reduce the levels of GluA1-AMPAR in the PFC and hippocampus, but elevate its levels in NAc (Yang et al., 2016). Furthermore, stress paradigms that impair hippocampal LTP have been found to facilitate amygdala LTP (Vouimba et al., 2004; Suvrathan et al., 2014). This implies that there may be important region-specific differences in AMPAR regulation and plasticity that need to be taken into account in the development of therapeutics.

Interestingly, increasing evidence suggests that stress facilitates the development of drug addiction. This may be attributed, at least in part, to stress-induced changes in synaptic plasticity (Lo Iacono et al., 2018; McGrath and Briand, 2019). Indeed, drugs of abuse and stress trigger common plasticity mechanisms in midbrain dopamine neurons. Acute stress and *in vivo* administration of drugs of abuse with different molecular mechanisms both enhanced the AMPAR/NMDAR EPSC ratio at excitatory synapses onto midbrain dopaminergic neurons (Saal et al., 2003). Another study found that early stressors such as repeated maternal separation increased TNF levels in the PFC and NAc while reducing GluA2 levels in male but not female rats. Maternally separated male rats display a greater preference for a cocaine-associated context, which was reversed by the TNF inhibitor XPro 1595 through normalizing TNF and GluA2 levels (Ganguly et al., 2019). Notably, chronic stress in humans is associated with higher rates of depression or depressive episodes (Kendler et al., 1999). Thus, the implications of these findings might extend to depression.

### Chronic Pain

Chronic pain conditions often have a psychological component in the form of a persistent sensory memory of the pain state, associated with fear, anxiety, and cognitive dysfunction. LTP and LTD in the dorsal horn of the spinal cord and cortical areas, including the anterior cingulate cortex (ACC), are increasingly thought to underlie chronic pain (Bliss et al., 2016). In particular, evidence from genetic and pharmacological studies indicates that the recruitment of GluA1-AMPARs to the postsynaptic membrane contributes to the expression of NMDAR-dependent LTP in the ACC (Toyoda et al., 2007, 2009; Xu et al., 2008; Li et al., 2010), the pathogenesis of chronic inflammation and neuropathic pain (Xu et al., 2008; Li et al., 2010), and chronic visceral pain (Liu et al., 2015; Wang et al., 2015). Analgesic effects were obtained by inhibiting AMPAR-mediated responses or reducing the expression of postsynaptic LTP in the ACC (Li et al., 2010; Chen et al., 2014; Liu et al., 2015; Wang et al., 2015; Zhuo, 2019).

### Epilepsy

Epilepsy is a neurological disorder characterized by recurrent and unprovoked seizures, reflecting episodic abnormal synchronized electrical activity in cerebral neuronal networks (Rogawski, 2013). Imbalanced excitatory and inhibitory synaptic transmission is thought to contribute to epilepsy pathogenesis (Bonansco and Fuenzalida, 2016). An elevation of hippocampal AMPAR levels has been reported in both temporal lobe epilepsy (TLE) patients and several animal epilepsy models (Mathern et al., 1998; Lopes et al., 2013). This is supported by a positron emission tomography (PET) tracer study of AMPARs ([11C]K-2), showing that [11C]K-2 uptake is increased in the epileptogenic focus of patients with mesial TLE (Miyazaki et al., 2020). Therapeutic strategies inhibiting AMPA-mediated currents, e.g., AMPAR antagonists, have been developed to treat epilepsy. AMPAR antagonists have been shown to alleviate epileptiform activity in *in vitro* models and confer protection from seizures in many animal seizure models (Rogawski, 2013). In particular, a selective non-competitive AMPAR antagonist, perampanel, has been clinically used to treat patients with partial-onset and tonic-clonic seizures (French et al., 2015a,b; Piña-Garza et al., 2020).

### Summary of Addiction, Stress, Chronic Pain, and Epilepsy Models

Taken together, the available data suggest that region- and subunit-specific pathological potentiation of AMPAR-mediated synaptic transmission is a common feature of animal models of addiction, stress, chronic pain, and epilepsy models (Figure 1, bottom left panel). In these models, experimental inhibition of potentiated AMPAR-mediated transmission, eg. using AMPAR antagonists or reversal of enhanced synaptic strength by regulating AMPAR trafficking and redistribution was able to mitigate addictive behavior, chronic pain, and epileptic seizures. In contrast, in the following sections, we review a set of disorders that are characterized by an impaired LTP or enhanced LTD, which has been associated with cognitive deficits.

### Huntington’s Disease (HD)

Huntington’s disease (HD) is an autosomal dominant inherited neurodegenerative disease, clinically characterized by cognitive deficits, psychiatric disturbance, and motor dysfunction. HD is caused by a mutated form of the huntingtin gene and the resulting mutant huntingtin protein (Saudou and Humbert, 2016). Increasing evidence suggests that cognitive and psychiatric disturbances occur in HD gene carriers and HD mouse models well before classical neuropathology or the onset of motor symptom, suggesting that the initial development of the disease results from a cellular dysfunction rather than a loss of neurons (Lemiere et al., 2004; Solomon et al., 2008). Various transgenic and knock-in HD mouse models exhibit impaired hippocampal LTP at the pre- or early-symptomatic stage (Hodgson et al., 1999; Murphy et al., 2000; Zhang et al., 2018). Consistently, behavioral studies reveal the deterioration of hippocampal-associated spatial memory in distinct HD murine models and patients (Chan et al., 2014; Majerová et al., 2012). Recent work has shown that AMPAR surface diffusion is dramatically increased in hippocampal neurons from HD rodent models. This was attributed to deficient brain-derived neurotrophic factor (BDNF)–Tropomyosin related kinase B (TrkB) signaling, which disrupted AMPAR diffusion trapping, i.e., the interaction between transmembrane AMPA receptor regulatory proteins (TARPs) and the PDZ-domain scaffold protein PSD95 (Zhang et al., 2018). The antidepressant tianeptine improved BDNF synthesis and intracellular transport, reversed AMPAR surface diffusion, and restored LTP and hippocampus-dependent memory in different HD mouse models (Zhang et al., 2018). AMPAR positive allosteric modulators (AMPAkines) have also been shown to rescue the deficits in synaptic plasticity and memory in HD mouse models, possibly via upregulating BDNF (Simmons et al., 2009, 2011).

### Alzheimer’s Disease (AD)

Alzheimer’s disease (AD) is a progressive neurodegenerative disease, clinically characterized by early memory deficits and progressive loss of higher cognitive functions. The causes of AD are unclear, but amyloid-β protein (Aβ) and tau are thought to play a central role in the etiology and pathogenesis. Extracellular amyloid plaques (composed of Aβ peptides) and intraneuronal neurofibrillary tangles (composed of tau) are pathological hallmarks of AD (Bloom, 2014). However, increasing evidence suggests that AD begins with synaptic failure before overt neuronal degeneration, which may contribute to the early memory impairments (Selkoe, 2002; Luscher and Huber, 2010; Opazo et al., 2018).

Impaired NMDAR-dependent hippocampal LTP or enhanced LTD are observed in various transgenic AD animal models (Mango et al., 2019). Tau protein appears to be involved in synaptic removal of AMPARs, as clustering of tau fibrils reduced synaptic abundance of AMPARs (Shrivastava et al., 2019). Moreover, Tau protein phosphorylation was involved in a ketamine-induced reduction in surface expression of AMPARs in hippocampal neurons (Li et al., 2019). Besides, mGluR-LTD induced by soluble Aβ oligomers is also thought to contribute to the mental decline in AD (Luscher and Huber, 2010). This appears to result from enhanced GluA2/GluA3-AMPAR endocytosis by Aβ oligomers (Jurado, 2018), which may require the participation of PICK1 (protein interacting with C kinase 1; Alfonso et al., 2014). Of particular note, Aβ-induced synaptic removal of AMPAR *via* endocytosis is necessary and sufficient to induce spine loss (Hsieh et al., 2006), which is in line with the role of GluA2-AMPAR in promoting dendritic spine formation and growth in cultured hippocampal neurons (Saglietti et al., 2007). This suggests that the dysregulation of AMPAR trafficking may have both adverse functional and structural consequences. Moreover, overexpression of the amyloid-precursor protein (APP) or exposure to Aβ oligomers resulted in abnormal enhancement of AMPAR surface diffusion *via* the activation of GluN2B-containing NMDARs (Opazo et al., 2018). Immobilizing AMPARs by crosslinking methods fully rescued spine loss induced by oligomeric Aβ (Opazo et al., 2018). These lines of evidence suggest that targeting of AMPAR trafficking may represent a new therapeutic avenue in AD and HD.

### Parkinson’s Disease (PD)

Parkinson’s disease (PD) is another neurodegenerative disorder associated with loss of dopaminergic neurons of the substantia nigra projection to the striatum, of major importance for motor control. The cause of PD is unknown but is believed to involve both genetic and environmental factors (Kalia and Lang, 2015). PD is not only characterized by motor symptoms but also non-motor symptoms, including cognitive impairment (Bernal-Pacheco et al., 2012; Modugno et al., 2013). The cognitive decline and dementia in PD have been associated with hippocampal dysfunction (Svenningsson et al., 2012; Calabresi et al., 2013; Cosgrove et al., 2015). For example, mutations in PD-associated E3 ubiquitin ligase Parkin have been associated with juvenile-onset PD. Parkin regulates synaptic AMPAR endocytosis *via* its binding and retention of the postsynaptic scaffold protein, homer (Cortese et al., 2016; Zhu et al., 2018). The four common Parkin point mutations (T240M, R275W, R334C, G430D; Zhu et al., 2018) or Parkin knock-down (Cortese et al., 2016) impaired this capacity, reduced surface expression of GluA1- and GluA2-AMPARs, and disrupted glutamatergic synaptic transmission in hippocampal neurons. It should be noted that Parkin is primarily involved in mitochondrial homeostasis (McWilliams and Muqit, 2017). Mitochondria, as energy centers and calcium buffer organelles, may play an important role in the regulation of synaptic plasticity (Todorova and Blokland, 2017). Moreover, PD dementia is considered a convergence of α-synuclein, tau, and Aβ pathologies (Irwin et al., 2013; Shrivastava et al., 2019). As discussed in the AD section, both tau and Aβ appear to be involved in the removal of synaptic AMPARs (Jurado, 2018; Li et al., 2019; Shrivastava et al., 2019). Thus, PD-related dementia might involve dysregulation of AMPAR trafficking induced by Aβ and tau.

In addition to PD dementia, the motor deficits of PD have also been partially attributed to aberrations in AMPAR plasticity. There is an imbalance in glutamatergic signaling between the direct pathway spiny projection neurons (dSPNs) and indirect pathway SPNs (iSPNs), with LTP (hyper AMPAR signaling) found in iSPNs while LTD (hypo AMPAR signaling) found in dSPNs (Shen et al., 2008; Fieblinger et al., 2014; Shields et al., 2017). In general, activity in the direct-pathway neurons would promote appropriate actions while the indirect-pathway suppresses unnecessary actions or movements. Potentiation or loss of inhibition in the indirect-pathway is thought to contribute to motor dysfunction (Luscher and Huber, 2010; Shields et al., 2017). In support of this idea, the AMPAR antagonist, YM90K, was specifically delivered to the indirect-pathway through the DART (drugs acutely restricted by tethering) technique, that used HaloTag to capture and tether drugs to the cell surface. The treatment profoundly ameliorated motor deficits, such as akinesia in PD animal models (Shields et al., 2017).

### Fragile X Syndrome (FXS)

Fragile X syndrome (FXS) is a neurodevelopmental disorder. It is the most common inherited cause of intellectual disability and a prevalent genetic cause of autism spectrum disorder (ASD; Cheng et al., 2017). FXS results from loss-of-function mutations in fragile X mental retardation protein (FMRP), an RNA-binding protein that regulates local translation of a subset of mRNAs at both presynaptic and postsynaptic locations in response to mGluR activation (Luscher and Huber, 2010). One primary consequence of FMRP loss is the enhancement of mGluR-LTD, which depends on GluA1-AMPAR endocytosis (Luscher and Huber, 2010; Cheng et al., 2017). *Fmr*1 knockout mice exhibit enhancement of mGluR-LTD both in the cerebellum and hippocampus (Luscher and Huber, 2010). Various mGluR5 antagonists have entered clinical trials (Berry-Kravis et al., 2018). This strategy aims at normalizing multiple cellular processes including LTD by targeting mGluRs rather than by directly interfering with ionotropic glutamate receptors, which have crucial physiological functions. However, the clinical efficacy of mGluR5 antagonists remains to be determined.

Impaired LTP in *Fmr*1 knockout mice has also been reported in multiple brain regions, such as the hippocampus (Lauterborn et al., 2007; Hu et al., 2008), anterior piriform cortex (Larson et al., 2005), deep-layer visual neocortex (Wilson and Cox, 2007), and ACC (Zhao et al., 2005; Wang et al., 2008). Restoring the synaptic delivery of GluA1-containing AMPARs by enhancing Ras-PI3K-Akt signaling rescued LTP in *Fmr1* KO mice (Hu et al., 2008; Lim et al., 2014). This indicates that targeting of AMPAR trafficking may be a potential therapeutic strategy in FXS.

### Summary of AD, HD, PD, and FXS Models

Taken together, the impairment in hippocampal or cortical LTP or the enhancement in LTD has been associated with behavioral and cognitive deficits in animal models of AD, HD, PD, and FXS (Figure 1, bottom right panel). Enhancing AMPAR diffusion trapping mechanisms (such as with tianeptine), blocking endocytosis (such as with mGluR5 antagonists), or enhancing AMPAR function (such as with AMPAR positive allosteric modulators) have exhibited therapeutic effects, such as reversal of synaptic plasticity and memory defects in animal models of AD, HD and FXS. However, the effects of these strategies in human patients remain to be determined. Brain region- and AMPAR subunit-specific treatment is needed.

## Discussion

While dysregulation of AMPAR synaptic plasticity is emerging as a point of convergence in multiple pathological pathways across several brain disorders, it is unlikely to be the major driver of pathology. Indeed, each brain disorder has a distinct and complex etiology and pathogenesis. Rather, the pathological impact on the excitatory synapse directly influences the expression of the disorders or associated symptoms, and these effects are reversible. Targeting a converging point of multiple pathological pathways, especially in early stages of diseases, such as HD and AD, may be more efficient than targeting any individual pathway. In addition, the pathological potentiation and weakening of synaptic strength both adversely impact the plastic range of the synapse and thus may hamper further plasticity (metaplasticity). Therefore, strategies that can preserve the plastic range of the synapse, eg. regulating AMPAR trafficking, will be beneficial. In particular, modulating AMPAR trafficking leads to AMPAR redistribution without perturbing AMPAR function, which is a great advantage.

Small interfering peptides that target protein-protein interactions are attracting increasing attention as potential therapeutics, partly due to their high binding specificity and affinity, and minimal off-target effects (Fosgerau and Hoffmann, 2015; Havasi et al., 2017). Interfering peptides targeting AMPAR endocytosis were shown effective in preventing the expression of D-amphetamine-induced behavioral sensitization and facilitating the extinction of morphine-induced conditioned place preference in rodent models of drug addiction (Brebner et al., 2005; Dias et al., 2012). We propose that peptides disrupting the diffusion trapping mechanism of AMPARs are an alternative strategy. Though promising, the therapeutic application of cell-penetrating peptides in humans remains challenging (Fosgerau and Hoffmann, 2015; Havasi et al., 2017). As an alternative, aptamers are oligonucleotide molecules that bind to a specific target molecule. For example, attempts have made to design RNA aptamers as AMPAR antagonists (Huang and Niu, 2019). However, there is still a long way to go to translate them into drug options.

A better understanding of the molecular mechanism and dynamics of AMPAR-mediated plasticity may help in the further identification of therapeutic targets. For dissecting mechanisms, the use of light-sensitive proteins allows fast and reversible manipulation of protein targets with high spatial-temporal precision (Paoletti et al., 2019). The development of optogenetic and optopharmacological tools may help to elucidate the spatial-temporal regulation of AMPAR trafficking in specific cell types and circuits in animal models. For example, genetically-encoded protein photosensors such as LOVTRAP and dimeric Dronpa, have been recently developed (Wang et al., 2016; Zhou et al., 2017). LOVTRAP can be used for reversible light-induced protein dissociation. It requires attaching one of the Zdk/LOV2 pairs to the target protein, and the other to the membrane (Wang and Hahn, 2016). Thus, LOVTRAP technology could serve to manipulate the synaptic anchoring of AMPARs by controlling the interaction between a membrane TARP and a PDZ-containing protein in the PSD. Another example is Dronpa, a reversibly photoswitchable fluorescent protein, which associates and dissociates in response to 400 nm and 500 nm illumination, respectively (Zhou et al., 2017). A generalizable method for optical control of kinases has been recently reported. Photoswitchable kinases, such as psRaf1, psMEK1, psMEK2, and psCDK5, have been successfully generated by attaching two photoswitchable dimeric Dronpa (pdDronpa) domains in the kinase functional domain. The light switch enables the caging and uncaging of the core kinase domain with high temporal and spatial precision (Zhou et al., 2017). This method could be used to elucidate local, synaptic regulation of AMPARs by protein kinases. Furthermore, a study successfully used a freely diffusible photoswitchable quinoxaline-2,3-dione, an antagonist selective for AMPARs, to control action potential firing optically (Barber et al., 2017). Another study developed a technique to inactivate synaptic GluA1 AMPARs *in vivo* using chromophore-assisted light inactivation and erased acquired fear memory in the animals (Takemoto et al., 2017). Thus, optogenetics and optopharmacology emerge as powerful tools for manipulating and interrogating synaptic plasticity with high spatial-temporal precision. Because light does not penetrate tissue easily, applying optogenetic tools in living animals often requires the implantation of invasive optical fibers into the brain. This has limited their applications in humans. Recently, an ultra-sensitive light-responsive molecule, SOUL, has been developed. Once engineered in the neurons inside the brain of mice and monkeys, the neurons can be turned on and off by illumination from outside of the head (Gong et al., 2020). Such non-invasive approaches hold promise for therapeutic optogenetics.

## Author Contributions

HZ and CB researched data for the article, wrote the article and reviewed and/or edited the manuscript before submission.

## Conflict of Interest

The authors declare that the research was conducted in the absence of any commercial or financial relationships that could be construed as a potential conflict of interest.

## References

[B1] Alfonso S. Kessels H. W. Banos C. C. Chan T. R. Lin E. T. Kumaravel G.. (2014). Synapto-depressive effects of amyloid β require PICK1. Eur. J. Neurosci. 39, 1225–1233. 10.1111/ejn.12499 24713001 PMC3983572

[B2] Barber D. M. Liu S. A. Gottschling K. Sumser M. Hollmann M. Trauner D. (2017). Optical control of AMPA receptors using a photoswitchable quinoxaline-2,3-dione antagonist. Chem. Sci. 8, 611–615. 10.1039/c6sc01621a 28451208 PMC5358534

[B3] Bernal-Pacheco O. Limotai N. Go C. L. Fernandez H. H. (2012). Nonmotor manifestations in Parkinson disease. Neurologist 18, 1–16. 10.1097/nrl.0b013e31823d7abb 22217609

[B4] Berry-Kravis E. M. Lindemann L. Jonch A. E. Apostol G. Bear M. F. Carpenter R. L.. (2018). Drug development for neurodevelopmental disorders: lessons learned from fragile X syndrome. Nat. Rev. Drug Discov. 17, 280–299. 10.1038/nrd.2017.221 29217836 PMC6904225

[B5] Bliss T. V. Collingridge G. L. Kaang B. K. Zhuo M. (2016). Synaptic plasticity in the anterior cingulate cortex in acute and chronic pain. Nat. Rev. Neurosci. 17, 485–496. 10.1038/nrn.2016.68 27307118

[B6] Bloom G. S. (2014). Amyloid-β and tau: the trigger and bullet in Alzheimer disease pathogenesis. JAMA Neurol. 71, 505–508. 10.1001/jamaneurol.2013.5847 24493463 PMC12908160

[B7] Bonansco C. Fuenzalida M. (2016). Plasticity of hippocampal excitatory-inhibitory balance: missing the synaptic control in the epileptic brain. Neural Plast. 2016:8607038. 10.1155/2016/8607038 27006834 PMC4783563

[B8] Brebner K. Wong T. P. Liu L. Liu Y. Campsall P. Gray S.. (2005). Nucleus accumbens long-term depression and the expression of behavioral sensitization. Science 310, 1340–1343. 10.1126/science.1116894 16311338

[B9] Calabresi P. Castrioto A. Di Filippo M. Picconi B. (2013). New experimental and clinical links between the hippocampus and the dopaminergic system in Parkinson’s disease. Lancet Neurol. 12, 811–821. 10.1016/s1474-4422(13)70118-2 23867199

[B10] Chan A. W. Xu Y. Jiang J. Rahim T. Zhao D. Kocerha J.. (2014). A two years longitudinal study of a transgenic Huntington disease monkey. BMC Neurosci. 15:36. 10.1186/1471-2202-15-36 24581271 PMC4015530

[B11] Chen T. Wang W. Dong Y. L. Zhang M. M. Wang J. Koga K.. (2014). Postsynaptic insertion of AMPA receptor onto cortical pyramidal neurons in the anterior cingulate cortex after peripheral nerve injury. Mol. Brain 7:76. 10.1186/s13041-014-0076-8 25359681 PMC4221704

[B12] Cheng G. R. Li X. Y. Xiang Y. D. Liu D. McClintock S. M. Zeng Y. (2017). The implication of AMPA receptor in synaptic plasticity impairment and intellectual disability in fragile X syndrome. Physiol. Res. 66, 715–727. 10.33549/physiolres.933473 28730825

[B13] Choi F. Y. Ahn S. Wang Y. T. Phillips A. G. (2014). Interference with AMPA receptor endocytosis: effects on behavioural and neurochemical correlates of amphetamine sensitization in male rats. J. Psychiatry Neurosci. 39, 189–199. 10.1503/jpn.120257 24290077 PMC3997604

[B14] Choquet D. (2018). Linking nanoscale dynamics of AMPA receptor organization to plasticity of excitatory synapses and learning. J. Neurosci. 38, 9318–9329. 10.1523/jneurosci.2119-18.2018 30381423 PMC6705996

[B15] Conrad K. L. Tseng K. Y. Uejima J. L. Reimers J. M. Heng L. J. Shaham Y.. (2008). Formation of accumbens GluR2-lacking AMPA receptors mediates incubation of cocaine craving. Nature 454, 118–121. 10.1038/nature06995 18500330 PMC2574981

[B16] Cooper S. Robison A. J. Mazei-Robison M. S. (2017). Reward circuitry in addiction. Neurotherapeutics 14, 687–697. 10.1007/s13311-017-0525-z 28324454 PMC5509624

[B17] Cortese G. P. Zhu M. Williams D. Heath S. Waites C. L. (2016). Parkin deficiency reduces hippocampal glutamatergic neurotransmission by impairing AMPA receptor endocytosis. J. Neurosci. 36, 12243–12258. 10.1523/jneurosci.1473-16.2016 27903732 PMC5148221

[B18] Cosgrove J. Alty J. E. Jamieson S. (2015). Cognitive impairment in Parkinson’s disease. Postgrad. Med. J. 91, 212–220. 10.1136/postgradmedj-2015-133247 25814509

[B19] Dias C. Wang Y. T. Phillips A. G. (2012). Facilitated extinction of morphine conditioned place preference with Tat-GluA2(3Y) interference peptide. Behav. Brain Res. 233, 389–397. 10.1016/j.bbr.2012.05.026 22633960

[B20] Fieblinger T. Graves S. M. Sebel L. E. Alcacer C. Plotkin J. L. Gertler T. S.. (2014). Cell type-specific plasticity of striatal projection neurons in parkinsonism and L-DOPA-induced dyskinesia. Nat. Commun. 5:5316. 10.1038/ncomms6316 25360704 PMC4431763

[B21] Fosgerau K. Hoffmann T. (2015). Peptide therapeutics: current status and future directions. Drug Discov. Today 20, 122–128. 10.1016/j.drudis.2014.10.003 25450771

[B22] French J. A. Gil-Nagel A. Malerba S. Kramer L. Kumar D. Bagiella E. (2015a). Time to prerandomization monthly seizure count in perampanel trials: a novel epilepsy endpoint. Neurology 84, 2014–2020. 10.1212/wnl.0000000000001585 25878175 PMC4442101

[B23] French J. A. Krauss G. L. Wechsler R. T. Wang X. F. DiVentura B. Brandt C.. (2015b). Perampanel for tonic-clonic seizures in idiopathic generalized epilepsy A randomized trial. Neurology 85, 950–957. 10.1212/WNL.0000000000001930 26296511 PMC4567458

[B24] Ganguly P. Honeycutt J. A. Rowe J. R. Demaestri C. Brenhouse H. C. (2019). Effects of early life stress on cocaine conditioning and AMPA receptor composition are sex-specific and driven by TNF. Brain Behav. Immun. 78, 41–51. 10.1016/j.bbi.2019.01.006 30654007 PMC6488364

[B25] Gong X. Mendoza-Halliday D. Ting J. T. Kaiser T. Sun X. Bastos A. M.. (2020). An ultra-sensitive step-function opsin for minimally invasive optogenetic stimulation in mice and macaques. Neuron [Epub ahead of print]. 10.1016/j.neuron.2020.03.032 32353253 PMC7351618

[B26] Good C. H. Lupica C. R. (2010). Afferent-specific AMPA receptor subunit composition and regulation of synaptic plasticity in midbrain dopamine neurons by abused drugs. J. Neurosci. 30, 7900–7909. 10.1523/jneurosci.1507-10.2010 20534838 PMC2900154

[B27] Groc L. Choquet D. Chaouloff F. (2008). The stress hormone corticosterone conditions AMPAR surface trafficking and synaptic potentiation. Nat. Neurosci. 11, 868–870. 10.1038/nn.2150 18622402

[B28] Havasi A. Lu W. Cohen H. T. Beck L. Wang Z. Igwebuike C.. (2017). Blocking peptides and molecular mimicry as treatment for kidney disease. Am. J. Physiol. Renal. Physiol. 312, F1016–F1025. 10.1152/ajprenal.00601.2015 27654896 PMC5495884

[B29] Hodgson J. G. Agopyan N. Gutekunst C. A. Leavitt B. R. LePiane F. Singaraja R.. (1999). A YAC mouse model for Huntington’s disease with full-length mutant huntingtin, cytoplasmic toxicity and selective striatal neurodegeneration. Neuron 23, 181–192. 10.1016/s0896-6273(00)80764-3 10402204

[B30] Hou Y. Y. Cai Y. Q. Pan Z. Z. (2020). GluA1 in central amygdala promotes opioid use and reverses inhibitory effect of pain. Neuroscience 426, 141–153. 10.1016/j.neuroscience.2019.11.032 31863796 PMC6941469

[B31] Hsieh H. Boehm J. Sato C. Iwatsubo T. Tomita T. Sisodia S.. (2006). AMPAR removal underlies Aβ-induced synaptic depression and dendritic spine loss. Neuron 52, 831–843. 10.1016/j.neuron.2006.10.035 17145504 PMC1850952

[B32] Hu H. Qin Y. Bochorishvili G. Zhu Y. van Aelst L. Zhu J. J. (2008). Ras signaling mechanisms underlying impaired GluR1-dependent plasticity associated with fragile X syndrome. J. Neurosci. 28, 7847–7862. 10.1523/jneurosci.1496-08.2008 18667617 PMC2553221

[B33] Hu H. Real E. Takamiya K. Kang M. G. Ledoux J. Huganir R. L.. (2007). Emotion enhances learning *via* norepinephrine regulation of AMPA-receptor trafficking. Cell 131, 160–173. 10.1016/j.cell.2007.09.017 17923095

[B35] Huang Z. Niu L. (2019). Developing RNA aptamers for potential treatment of neurological diseases. Future Med. Chem. 11, 551–565. 10.4155/fmc-2018-0364 30912676 PMC6661927

[B34] Huang Y. H. Schluter O. M. Dong Y. (2015). Silent synapses speak up: updates of the neural rejuvenation hypothesis of drug addiction. Neuroscientist 21, 451–459. 10.1177/1073858415579405 25829364 PMC4675132

[B36] Irwin D. J. Lee V. M. Trojanowski J. Q. (2013). Parkinson’s disease dementia: convergence of α-synuclein, tau and amyloid-β pathologies. Nat. Rev. Neurosci. 14, 626–636. 10.1038/nrn3549 23900411 PMC4017235

[B37] Jurado S. (2018). AMPA receptor trafficking in natural and pathological aging. Front. Mol. Neurosci. 10:446. 10.3389/fnmol.2017.00446 29375307 PMC5767248

[B38] Kakegawa W. Katoh A. Narumi S. Miura E. Motohashi J. Takahashi A.. (2018). Optogenetic control of synaptic AMPA receptor endocytosis reveals roles of LTD in motor learning. Neuron 99, 985.e6–998.e6. 10.1016/j.neuron.2018.07.034 30122381

[B39] Kalia L. V. Lang A. E. (2015). Parkinson’s disease. Lancet 386, 896–912. 10.1016/S0140-6736(14)61393-3 25904081

[B40] Karst H. Berger S. Turiault M. Tronche F. Schutz G. Joels M. (2005). Mineralocorticoid receptors are indispensable for nongenomic modulation of hippocampal glutamate transmission by corticosterone. Proc. Natl. Acad. Sci. U S A 102, 19204–19207. 10.1073/pnas.0507572102 16361444 PMC1323174

[B41] Karst H. Joëls M. (2005). Corticosterone slowly enhances miniature excitatory postsynaptic current amplitude in mice CA1 hippocampal cells. J. Neurophysiol. 94, 3479–3486. 10.1152/jn.00143.2005 16033944

[B42] Kendler K. S. Karkowski L. M. Prescott C. A. (1999). Causal relationship between stressful life events and the onset of major depression. Am. J. Psychiatry 156, 837–841. 10.1176/ajp.156.6.837 10360120

[B43] Kim J. J. Diamond D. M. (2002). The stressed hippocampus, synaptic plasticity and lost memories. Nat. Rev. Neurosci. 3, 453–462. 10.1038/nrn849 12042880

[B44] Krugers H. J. Hoogenraad C. C. Groc L. (2010). Stress hormones and AMPA receptor trafficking in synaptic plasticity and memory. Nat. Rev. Neurosci. 11, 675–681. 10.1038/nrn2913 20820185

[B45] Larson J. Jessen R. E. Kim D. Fine A. K. du Hoffmann J. (2005). Age-dependent and selective impairment of long-term potentiation in the anterior piriform cortex of mice lacking the fragile X mental retardation protein. J. Neurosci. 25, 9460–9469. 10.1523/jneurosci.2638-05.2005 16221856 PMC6725716

[B46] Lauterborn J. C. Rex C. S. Kramar E. Chen L. Y. Pandyarajan V. Lynch G.. (2007). Brain-derived neurotrophic factor rescues synaptic plasticity in a mouse model of fragile X syndrome. J. Neurosci. 27, 10685–10694. 10.1523/jneurosci.2624-07.2007 17913902 PMC6672822

[B47] Lee B. R. Ma Y. Y. Huang Y. H. Wang X. Otaka M. Ishikawa M.. (2013). Maturation of silent synapses in amygdala-accumbens projection contributes to incubation of cocaine craving. Nat. Neurosci. 16, 1644–1651. 10.1038/nn.3533 24077564 PMC3815713

[B48] Lemiere J. Decruyenaere M. Evers-Kiebooms G. Vandenbussche E. Dom R. (2004). Cognitive changes in patients with Huntington’s disease (HD) and asymptomatic carriers of the HD mutation–a longitudinal follow-up study. J. Neurol. 251, 935–942. 10.1007/s00415-004-0461-9 15316797

[B50] Li Y. Ding R. Ren X. Wen G. Dong Z. Yao H.. (2019). Long-term ketamine administration causes Tau protein phosphorylation and Tau protein-dependent AMPA receptor reduction in the hippocampus of mice. Toxicol. Lett. 315, 107–115. 10.1016/j.toxlet.2019.08.023 31470060

[B49] Li X. Y. Ko H. G. Chen T. Descalzi G. Koga K. Wang H.. (2010). Alleviating neuropathic pain hypersensitivity by inhibiting PKMzeta in the anterior cingulate cortex. Science 330, 1400–1404. 10.1126/science.1191792 21127255

[B51] Lim C. S. Hoang E. T. Viar K. E. Stornetta R. L. Scott M. M. Zhu J. J. (2014). Pharmacological rescue of Ras signaling, GluA1-dependent synaptic plasticity and learning deficits in a fragile X model. Genes Dev. 28, 273–289. 10.1101/gad.232470.113 24493647 PMC3923969

[B52] Liu S. B. Zhang M. M. Cheng L. F. Shi J. Lu J. S. Zhuo M. (2015). Long-term upregulation of cortical glutamatergic AMPA receptors in a mouse model of chronic visceral pain. Mol. Brain 8:76. 10.1186/s13041-015-0169-z 26585043 PMC4653882

[B53] Lo Iacono L. Catale C. Martini A. Valzania A. Viscomi M. T. Chiurchiu V.. (2018). From traumatic childhood to cocaine abuse: the critical function of the immune system. Biol. Psychiatry 84, 905–916. 10.1016/j.biopsych.2018.05.022 30029767

[B54] Lopes M. W. Soares F. M. de Mello N. Nunes J. C. Cajado A. G. de Brito D.. (2013). Time-dependent modulation of AMPA receptor phosphorylation and mRNA expression of NMDA receptors and glial glutamate transporters in the rat hippocampus and cerebral cortex in a pilocarpine model of epilepsy. Exp. Brain Res. 226, 153–163. 10.1007/s00221-013-3421-8 23392471

[B55] Loweth J. A. Scheyer A. F. Milovanovic M. LaCrosse A. L. Flores-Barrera E. Werner C. T.. (2014). Synaptic depression *via* mGluR1 positive allosteric modulation suppresses cue-induced cocaine craving. Nat. Neurosci. 17, 73–80. 10.1038/nn.3590 24270186 PMC3971923

[B56] Luscher C. Huber K. M. (2010). Group 1 mGluR-dependent synaptic long-term depression: mechanisms and implications for circuitry and disease. Neuron 65, 445–459. 10.1016/j.neuron.2010.01.016 20188650 PMC2841961

[B57] Ma T. Cheng Y. Roltsch Hellard E. Wang X. Lu J. Gao X.. (2018). Bidirectional and long-lasting control of alcohol-seeking behavior by corticostriatal LTP and LTD. Nat. Neurosci. 21, 373–383. 10.1038/s41593-018-0081-9 29434375 PMC5857235

[B58] Ma Y. Y. Lee B. R. Wang X. Guo C. Liu L. Cui R.. (2014). Bidirectional modulation of incubation of cocaine craving by silent synapse-based remodeling of prefrontal cortex to accumbens projections. Neuron 83, 1453–1467. 10.1016/j.neuron.2014.08.023 25199705 PMC4295617

[B59] Madayag A. C. Gomez D. Anderson E. M. Ingebretson A. E. Thomas M. J. Hearing M. C. (2019). Cell-type and region-specific nucleus accumbens AMPAR plasticity associated with morphine reward, reinstatement and spontaneous withdrawal. Brain Struct. Funct. 224, 2311–2324. 10.1007/s00429-019-01903-y 31201496 PMC6698404

[B60] Majerová V. Kalinčík T. Laczó J. Vyhnálek M. Hort J. Bojar M.. (2012). Disturbance of real space navigation in moderately advanced but not in early Huntington’s disease. J. Neurol. Sci. 312, 86–91. 10.1016/j.jns.2011.08.016 21875725

[B61] Makino H. Malinow R. (2009). AMPA receptor incorporation into synapses during LTP: the role of lateral movement and exocytosis. Neuron 64, 381–390. 10.1016/j.neuron.2009.08.035 19914186 PMC2999463

[B62] Mango D. Saidi A. Cisale G. Y. Feligioni M. Corbo M. Nistico R. (2019). Targeting synaptic plasticity in experimental models of Alzheimer’s disease. Front. Pharmacol. 10:778. 10.3389/fphar.2019.00778 31379566 PMC6646937

[B63] Marchi M. Grilli M. Pittaluga A. M. (2015). Nicotinic modulation of glutamate receptor function at nerve terminal level: a fine-tuning of synaptic signals. Front. Pharmacol. 6:89. 10.3389/fphar.2015.00089 25972809 PMC4413670

[B64] Mathern G. W. Pretorius J. K. Leite J. P. Kornblum H. I. Mendoza D. Lozada A.. (1998). Hippocampal AMPA and NMDA mRNA levels and subunit immunoreactivity in human temporal lobe epilepsy patients and a rodent model of chronic mesial limbic epilepsy. Epilepsy Res. 32, 154–171. 10.1016/s0920-1211(98)00048-5 9761317

[B65] McGrath A. G. Briand L. A. (2019). A potential role for microglia in stress- and drug-induced plasticity in the nucleus accumbens: a mechanism for stress-induced vulnerability to substance use disorder. Neurosci. Biobehav. Rev. 107, 360–369. 10.1016/j.neubiorev.2019.09.007 31550452 PMC6924541

[B66] McWilliams T. G. Muqit M. M. (2017). PINK1 and Parkin: emerging themes in mitochondrial homeostasis. Curr. Opin. Cell Biol. 45, 83–91. 10.1016/j.ceb.2017.03.013 28437683

[B67] Miyazaki T. Nakajima W. Hatano M. Shibata Y. Kuroki Y. Arisawa T.. (2020). Visualization of AMPA receptors in living human brain with positron emission tomography. Nat. Med. 26, 281–288. 10.1038/s41591-019-0723-9 31959988

[B68] Modugno N. Lena F. Di Biasio F. Cerrone G. Ruggieri S. Fornai F. (2013). A clinical overview of non-motor symptoms in Parkinson’s disease. Arch. Ital. Biol. 151, 148–168. 24873924

[B69] Murphy K. P. Carter R. J. Lione L. A. Mangiarini L. Mahal A. Bates G. P.. (2000). Abnormal synaptic plasticity and impaired spatial cognition in mice transgenic for exon 1 of the human Huntington’s disease mutation. J. Neurosci. 20, 5115–5123. 10.1523/jneurosci.20-13-05115.2000 10864968 PMC6772265

[B70] Nabavi S. Fox R. Proulx C. D. Lin J. Y. Tsien R. Y. Malinow R. (2014). Engineering a memory with LTD and LTP. Nature 511, 348–352. 10.1038/nature13294 24896183 PMC4210354

[B71] Opazo P. Choquet D. (2011). A three-step model for the synaptic recruitment of AMPA receptors. Mol. Cell. Neurosci. 46, 1–8. 10.1016/j.mcn.2010.08.014 20817097

[B72] Opazo P. Viana da Silva S. Carta M. Breillat C. Coultrap S. J. Grillo-Bosch D.. (2018). CaMKII metaplasticity drives Aβ oligomer-mediated synaptotoxicity. Cell Rep. 23, 3137–3145. 10.1016/j.celrep.2018.05.036 29898386 PMC6089247

[B73] Paoletti P. Ellis-Davies G. C. R. Mourot A. (2019). Optical control of neuronal ion channels and receptors. Nat. Rev. Neurosci. 20, 514–532. 10.1038/s41583-019-0197-2 31289380 PMC6703956

[B74] Pascoli V. Terrier J. Espallergues J. Valjent E. O’Connor E. C. Luscher C. (2014). Contrasting forms of cocaine-evoked plasticity control components of relapse. Nature 509, 459–464. 10.1038/nature13257 24848058

[B75] Penn A. C. Zhang C. L. Georges F. Royer L. Breillat C. Hosy E.. (2017). Hippocampal LTP and contextual learning require surface diffusion of AMPA receptors. Nature 549, 384–388. 10.1038/nature23658 28902836 PMC5683353

[B76] Piña-Garza J. E. Rosenfeld W. Saeki K. Villanueva V. Yoshinaga H. Patten A.. (2020). Efficacy and safety of adjunctive perampanel in adolescent patients with epilepsy: Post hoc analysis of six randomized studies. Epilepsy Behav. 104:106876. 10.1016/j.yebeh.2019.106876 31954998

[B77] Rogawski M. A. (2013). AMPA receptors as a molecular target in epilepsy therapy. Acta Neurol. Scand. Suppl. 197, 9–18. 10.1111/ane.12099 23480151 PMC4506648

[B78] Saal D. Dong Y. Bonci A. Malenka R. C. (2003). Drugs of abuse and stress trigger a common synaptic adaptation in dopamine neurons. Neuron 37, 577–582. 10.1016/s0896-6273(03)00021-7 12597856

[B79] Saglietti L. Dequidt C. Kamieniarz K. Rousset M. C. Valnegri P. Thoumine O.. (2007). Extracellular interactions between GluR2 and N-cadherin in spine regulation. Neuron 54, 461–477. 10.1016/j.neuron.2007.04.012 17481398

[B80] Saudou F. Humbert S. (2016). The biology of Huntingtin. Neuron 89, 910–926. 10.1016/j.neuron.2016.02.003 26938440

[B81] Scheyer A. F. Loweth J. A. Christian D. T. Uejima J. Rabei R. Le T.. (2016). AMPA receptor plasticity in accumbens core contributes to incubation of methamphetamine craving. Biol. Psychiatry 80, 661–670. 10.1016/j.biopsych.2016.04.003 27264310 PMC5050076

[B82] Selkoe D. J. (2002). Alzheimer’s disease is a synaptic failure. Science 298, 789–791. 10.1126/science.1074069 12399581

[B83] Shen W. Flajolet M. Greengard P. Surmeier D. J. (2008). Dichotomous dopaminergic control of striatal synaptic plasticity. Science 321, 848–851. 10.1126/science.1160575 18687967 PMC2833421

[B84] Shields B. C. Kahuno E. Kim C. Apostolides P. F. Brown J. Lindo S.. (2017). Deconstructing behavioral neuropharmacology with cellular specificity. Science 356:eaaj2161. 10.1126/science.aaj2161 28385956

[B85] Shrivastava A. N. Redeker V. Pieri L. Bousset L. Renner M. Madiona K.. (2019). Clustering of Tau fibrils impairs the synaptic composition of α3-Na^+^/K^+^-ATPase and AMPA receptors. EMBO J. 38:e99871. 10.15252/embj.201899871 30630857 PMC6356061

[B112] Simmons D. A. Rex C. S. Palmer L. Pandyarajan V. Fedulov V. Gall C. M.. (2009). Up-regulating BDNF with an ampakine rescues synaptic plasticity and memory in Huntington’s Disease knockin mice. Proc. Natl. Acad. Sci. U S A. 24, 4906–5011. 10.1073/pnas.0811228106 19264961 PMC2660722

[B113] Simmons D. A. Mehta R. A. Lauterborn J. C. Gall C. M. Lynch G. (2011). Brief ampakine treatements slow the progression of Huntington’s disease phenotypes in R6/2 mice. Nero. Bio. Dis. 41, 436–444. 10.1016/j.nbd.2010.10.015 20977939 PMC3014441

[B86] Solomon A. C. Stout J. C. Weaver M. Queller S. Tomusk A. Whitlock K. B.. (2008). Ten-year rate of longitudinal change in neurocognitive and motor function in prediagnosis Huntington disease. Mov. Disord. 23, 1830–1836. 10.1002/mds.22097 18785217 PMC2592091

[B87] Suvrathan A. Bennur S. Ghosh S. Tomar A. Anilkumar S. Chattarji S. (2014). Stress enhances fear by forming new synapses with greater capacity for long-term potentiation in the amygdala. Philos. Trans. R. Soc. Lond. B Biol. Sci. 369:20130151. 10.1098/rstb.2013.0151 24298153 PMC3843883

[B88] Svenningsson P. Westman E. Ballard C. Aarsland D. (2012). Cognitive impairment in patients with Parkinson’s disease: diagnosis, biomarkers and treatment. Lancet Neurol. 11, 697–707. 10.1016/s1474-4422(12)70152-7 22814541

[B89] Takemoto K. Iwanari H. Tada H. Suyama K. Sano A. Nagai T.. (2017). Optical inactivation of synaptic AMPA receptors erases fear memory. Nat. Biotechnol. 35, 38–47. 10.1038/nbt.3710 27918547

[B90] Tan K. R. Brown M. Labouebe G. Yvon C. Creton C. Fritschy J. M.. (2010). Neural bases for addictive properties of benzodiazepines. Nature 463, 769–774. 10.1038/nature08758 20148031 PMC2871668

[B91] Todorova V. Blokland A. (2017). Mitochondria and synaptic plasticity in the mature and aging nervous system. Curr. Neuropharmacol. 15, 166–173. 10.2174/1570159x14666160414111821 27075203 PMC5327446

[B92] Toyoda H. Wu L. J. Zhao M. G. Xu H. Zhuo M. (2007). Time-dependent postsynaptic AMPA GluR1 receptor recruitment in the cingulate synaptic potentiation. Dev. Neurobiol. 67, 498–509. 10.1002/dneu.20380 17443804

[B93] Toyoda H. Zhao M. G. Ulzhofer B. Wu L. J. Xu H. Seeburg P. H.. (2009). Roles of the AMPA receptor subunit GluA1 but not GluA2 in synaptic potentiation and activation of ERK in the anterior cingulate cortex. Mol. Pain 5:46. 10.1186/1744-8069-5-46 19664265 PMC2734546

[B94] Ungless M. A. Whistler J. L. Malenka R. C. Bonci A. (2001). Single cocaine exposure *in vivo* induces long-term potentiation in dopamine neurons. Nature 411, 583–587. 10.1038/35079077 11385572

[B95] Van den Oever M. C. Goriounova N. A. Li K. W. Van der Schors R. C. Binnekade R. Schoffelmeer A. N.. (2008). Prefrontal cortex AMPA receptor plasticity is crucial for cue-induced relapse to heroin-seeking. Nat. Neurosci. 11, 1053–1058. 10.1038/nn.2165 19160503

[B96] Vouimba R. M. Yaniv D. Diamond D. Richter-Levin G. (2004). Effects of inescapable stress on LTP in the amygdala versus the dentate gyrus of freely behaving rats. Eur. J. Neurosci. 19, 1887–1894. 10.1111/j.1460-9568.2004.03294.x 15078562

[B97] Wang H. Hahn K. M. (2016). LOVTRAP: a versatile method to control protein function with light. Curr. Protoc. Cell Biol. 73, 21.10.21–21.10.14. 10.1002/cpcb.12 27906450 PMC5137945

[B98] Wang H. Vilela M. Winkler A. Tarnawski M. Schlichting I. Yumerefendi H.. (2016). LOVTRAP: an optogenetic system for photoinduced protein dissociation. Nat. Methods 13, 755–758. 10.1038/nmeth.3926 27427858 PMC5137947

[B99] Wang H. Wu L. J. Kim S. S. Lee F. J. Gong B. Toyoda H.. (2008). FMRP acts as a key messenger for dopamine modulation in the forebrain. Neuron 59, 634–647. 10.1016/j.neuron.2008.06.027 18760699

[B100] Wang J. Zhang X. Cao B. Liu J. Li Y. (2015). Facilitation of synaptic transmission in the anterior cingulate cortex in viscerally hypersensitive rats. Cereb. Cortex 25, 859–868. 10.1093/cercor/bht273 24108805 PMC4379994

[B101] Wilson B. M. Cox C. L. (2007). Absence of metabotropic glutamate receptor-mediated plasticity in the neocortex of fragile X mice. Proc. Natl. Acad. Sci. U S A 104, 2454–2459. 10.1073/pnas.0610875104 17287348 PMC1892931

[B102] Wolf M. E. (2016). Synaptic mechanisms underlying persistent cocaine craving. Nat. Rev. Neurosci. 17, 351–365. 10.1038/nrn.2016.39 27150400 PMC5466704

[B103] Xu H. Wu L. J. Wang H. Zhang X. Vadakkan K. I. Kim S. S.. (2008). Presynaptic and postsynaptic amplifications of neuropathic pain in the anterior cingulate cortex. J. Neurosci. 28, 7445–7453. 10.1523/JNEUROSCI.1812-08.2008 18632948 PMC3844787

[B104] Yang B. Zhang J. C. Han M. Yao W. Yang C. Ren Q.. (2016). Comparison of R-ketamine and rapastinel antidepressant effects in the social defeat stress model of depression. Psychopharmacology 233, 3647–3657. 10.1007/s00213-016-4399-2 27488193 PMC5021744

[B105] Zhang H. Etherington L. A. Hafner A. S. Belelli D. Coussen F. Delagrange P.. (2013). Regulation of AMPA receptor surface trafficking and synaptic plasticity by a cognitive enhancer and antidepressant molecule. Mol. Psychiatry 18, 471–484. 10.1038/mp.2012.80 22733125 PMC3606944

[B106] Zhang H. Zhang C. Vincent J. Zala D. Benstaali C. Sainlos M.. (2018). Modulation of AMPA receptor surface diffusion restores hippocampal plasticity and memory in Huntington’s disease models. Nat. Commun. 9:4272. 10.1038/s41467-018-06675-3 30323233 PMC6189172

[B107] Zhao M. G. Toyoda H. Ko S. W. Ding H. K. Wu L. J. Zhuo M. (2005). Deficits in trace fear memory and long-term potentiation in a mouse model for fragile X syndrome. J. Neurosci. 25, 7385–7392. 10.1523/jneurosci.1520-05.2005 16093389 PMC6725289

[B108] Zhou X. X. Fan L. Z. Li P. Shen K. Lin M. Z. (2017). Optical control of cell signaling by single-chain photoswitchable kinases. Science 355, 836–842. 10.1126/science.aah3605 28232577 PMC5589340

[B109] Zhu M. Cortese G. P. Waites C. L. (2018). Parkinson’s disease-linked Parkin mutations impair glutamatergic signaling in hippocampal neurons. BMC Biol. 16:100. 10.1186/s12915-018-0567-7 30200940 PMC6130078

[B111] Zhuo M. (2019). Long-term cortical synaptic changes contribute to chronic pain and emotional disorders. Neurosci. Lett. 702, 66–70. 10.1016/j.neulet.2018.11.048 30503924

